# The Synthesis of Carbon Black-Loaded Pt Concave Nanocubes with High-Index Facets and Their Enhanced Electrocatalytic Properties toward Glucose Oxidation

**DOI:** 10.3390/nano12213761

**Published:** 2022-10-26

**Authors:** Xin Xu, Ze Ma, Zekun Su, Danqing Li, Xufeng Dong, Hao Huang, Min Qi

**Affiliations:** 1 Key Laboratory of Energy Materials and Devices (Liaoning Province), School of Materials Science and Engineering, Dalian University of Technology, Dalian 116024, China; 2 The Second Affiliated Hospital of Dalian Medical University, Dalian 116023, China

**Keywords:** platinum, high-index facets, carbon support, glucose oxidation, electrocatalysts

## Abstract

Catalysts with high catalytic activity and good stability are desirable in the electrocatalytic oxidation of glucose. Herein, Pt concave nanocubes with high-index facets (HIFs) supported by carbon black (Pt CNC/CB) are prepared through a hydrothermal method. The experimental results demonstrate that the peak current densities in different potential regions on the Pt CNC/CB anode are 0.22, 0.20, and 0.60 mA cm^−2^. The catalytic process of the glucose oxidation reaction is investigated in electrolytes with different pH values. Better stability is achieved by Pt CNC/CB than by Pt concave nanocubes (Pt CNCs). Abundant surface defects with low-coordinated atom numbers, such as steps, kinks, and edges, served as active sites in the electrocatalytic oxidation of glucose. With the addition of carbon black, the catalytic activity can be improved by facilitating the full exposure of the active surface defects on the HIFs of the Pt CNCs. Moreover, to address the aggregation of Pt CNCs, caused by the high surface energy of HIFs, the introduction of carbon material is an effective way to preserve the HIFs and thus enhance the stability of the catalyst. Hence, the prepared Pt CNC/CB electrocatalyst has great potential to be applied in the electrooxidation of glucose.

## 1. Introduction

As the main source of energy for human metabolism, glucose is generally present in body fluids. Many favorable features, including stability, safety, and abundance, make it a good choice of fuel for implantable glucose fuel cells [1,2,3]. Electricity can be converted by the oxidation of glucose at the anode and the reduction of oxygen at the cathode [4,5,6]. This kind of glucose fuel cell is a promising power supply for various implantable medical devices, such as pacemakers, sensors, artificial heart devices, and electrodes to treat neurological diseases [7]. It is regarded as an alternative to lithium batteries, which are difficult to miniaturize and are restricted by a limited lifetime [8]. However, the oxidation of glucose is a difficult step, which is affected by slow kinetics [9]. To overcome this problem, an efficient catalyst for glucose oxidation has been largely studied.

Abiotic catalysts, especially noble metals and their alloys, are suitable candidates that have good stability in the in vivo environment [10,11]. It is indicated that the electrocatalytic oxidation of glucose on platinum (Pt) single crystals is a structure-sensitive reaction [7]. The reactivity of Pt-based catalysts can be governed by varying the exposure of crystal facets, tuning the shapes, and controlling the sizes [12,13]. A large number of studies have demonstrated that nanocrystals (NCs) enclosed by high-index facets (HIFs) exhibit high catalytic properties due to the defect structures with low coordination atoms, such as steps, kinks, and edges [14,15,16], and Pt-based NCs with HIFs have been widely researched for many catalytic applications [17,18,19,20]. However, due to the active unsaturated atoms and the high surface energy, the phenomena of atom migration and aggregation can easily occur on the surface of Pt-based NCs enclosed by HIFs, which may result in surface restructuring and the attenuation of the surface activity [21]. Hence, maintaining the high activity and preserving the HIFs of Pt-based catalysts are key issues to be addressed. A suitable material that can provide a large surface area for Pt distribution is recommended to avoid aggregation.

Carbon materials as typical electrical conductivity supports have attracted extensive attention. The characteristic of the high specific surface area makes it an appropriate support for different catalysts [22,23]. Generally, loading Pt-based NCs on the carbon supports (graphene, carbon nanotubes, carbon black, etc.) is an effective strategy for enhancing the catalytic stability and promoting the dispersion of the catalyst [24,25,26,27,28,29]. Zhou et al. investigated the catalytic properties of high-index faceted Pt NCs supported on carbon black (HIF-Pt/C) for ethanol electrooxidation. The HIF-Pt/C catalysts exhibited 2–3 times higher electrocatalytic activity than the commercial Pt/C catalysts for ethanol oxidation reaction, which can be attributed to their high density of atomic steps [30]. Zhao et al. prepared three-dimensional (3D) flowerlike Pt nanoparticle clusters on multiwalled carbon nanotubes (MWCNTs) through an electrochemical method. The resulting Pt/MWCNTs in 3D morphology exhibited significantly higher electrocatalytic activity and better stability than the dispersive morphology in the glucose oxidation reaction and oxygen reduction reaction [31]. Although numerous studies on carbon-supported Pt-based catalysts with HIFs have been reported, to our knowledge, they have rarely been studied in the electrocatalytic oxidation of glucose.

In this study, Pt concave nanocubes (Pt CNCs) with HIFs supported by Vulcan XC-72R carbon black (CB) were synthesized as catalysts for the oxidation of glucose and labeled as Pt CNCs/CB. The surface morphology and structure of Pt CNC/CB were characterized. The catalytic performances in the glucose oxidation reaction were measured under neutral conditions. The effect of the typical Vulcan XC-72R CB support in Pt CNC/CB was evaluated. It is expected that the aggregation of Pt CNCs can be alleviated by the addition of a carbon support with a sufficient surface area. Therefore, the surface defect structures derived from the HIFs of Pt CNCs can be fully exposed, which are generally considered to be the active sites in catalytic reactions.

## 2. Materials and Methods

### 2.1. Chemicals

The Vulcan XC-72R carbon black (CB) was obtained from Cabot Inc., Boston, MA, USA. Polyvinylpyrrolidone (PVP), glycine, glucose, and hexachloroplatinic acid hexahydrate (H_2_PtCl_6_·6H_2_O) were purchased from Shanghai Macklin Biochemical Co., Ltd. (Shanghai, China). The phosphate-buffered saline (PBS, pH 7.4) solution was obtained from Beijing Solarbio Science & Technology Co., Ltd. (Beijing, China). All chemicals were of analytical grade and were used without further purification.

### 2.2. Synthesis of Pt CNC/CB Catalysts

**Figure 1:**
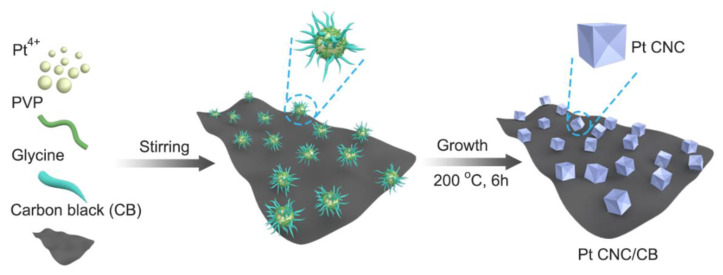
Schematic illustration of the synthesis of the Pt CNC/CB catalyst.

The Pt CNC/CB catalyst was synthesized by using a facile hydrothermal method (Figure 1). A specific amount of CB (3 mg), PVP (MW = 28,000, 800 mg), and glycine (150 mg) were added to deionized water with stirring. After that, 2 mL of H_2_PtCl_6_·6H_2_O solution (40 mM) was added to the above solution and stirred at room temperature for a few minutes. Then, the as-prepared homogeneous solution was transferred to a Teflon-lined stainless-steel autoclave. The sealed vessel was held at 200 °C for 6 h before it cooled down to room temperature. The products were separated by centrifugation at 10,000 rpm for 15 min and were then purified by washing with ethanol three times. 

### 2.3. Characterization

The surface morphology of the samples was characterized by transmission electron microscopy (TEM) and high-resolution transmission electron microscopy (HRTEM) on a Tecnai G2F30 STWIN (FEI Inc., Hillsboro, OR, USA), operating at an acceleration voltage of 200 kV. X-ray photoelectron spectroscopy (XPS) measurements were conducted on a Thermo ESCALAB 250 spectrometer (Waltham, MA, USA) using a monochromic Al Kα X-ray source. The functional group information was identified by Fourier transformed infrared (FT-IR) on a Thermo Fisher 6700 (Waltham, MA, USA).

### 2.4. Electrochemical Measurements

Electrochemical measurements including cyclic voltammetry (CV) and chronoamperometry (CA) were performed on an M204 electrochemical workstation (Metrohm Autolab, Utrecht, Netherlands). The CV tests were conducted in 0.1 M PBS (pH 7.4) solution with and without the addition of 50 mM glucose. The potential ranged from 0.079 to 1.679 V, and the scan rate was 20 mV s^−1^. Before the electrochemical measurements, the activation of the electrode was conducted by fast CV scanning from 0.042 to 1.242 V in a 0.5 M H_2_SO_4_ solution. The electrochemically active surface area (ECSA) of the catalyst was calculated based on the CV curves, measured in oxygen-free 0.5 M H_2_SO_4_ solution from 0.042 to 1.242 V at a scan rate of 50 mV s^−1^. The correlations between the CV scan rate, pH value, and the current density were investigated. CA measurement was performed at a potential of 1.279 V in 0.1 M PBS (pH 7.4) solution with the addition of 50 mM glucose. The ink for electrochemical measurements was prepared by adding the catalyst (5 mg) to ethanol (1 mL), followed by sonication for 30 min. A working electrode was prepared by loading the ink (3 μm) on a glassy carbon electrode (GCE) at 25 °C and then drying naturally. The saturated calomel electrode (SCE) and a Pt sheet (1 cm^2^) were used as the reference and counter electrodes, respectively. All of the potentials mentioned in this article were converted to the reversible hydrogen electrode (RHE) via the equation $E_{\mathrm{RHE}}=E_{\mathrm{SCE}}+0.242+0.0591\times \mathrm{pH}$.

## 3. Results and Discussion

### 3.1. Physicochemical Characterizations

**Figure 2:**
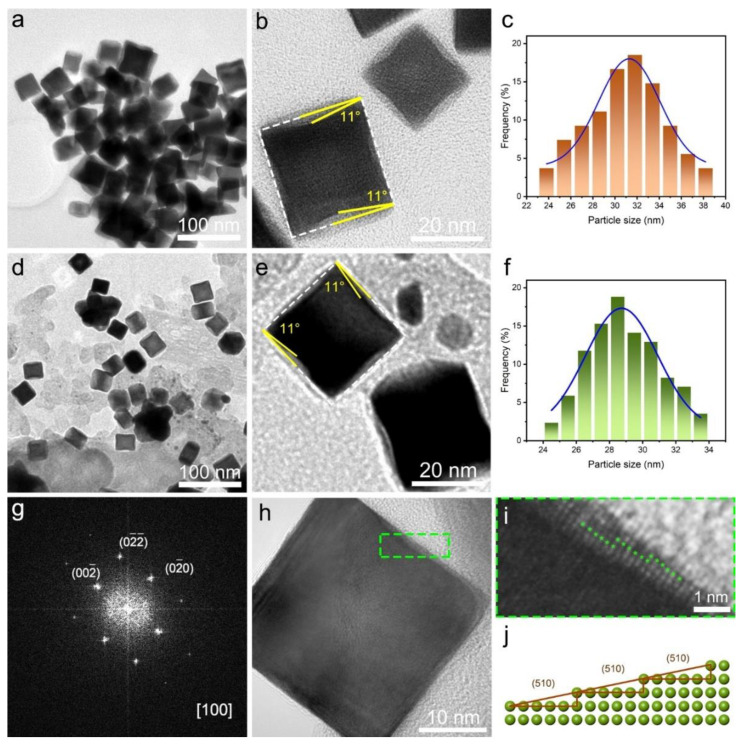
TEM, HRTEM images, and particle size distribution of Pt CNC (**a**–**c**), and Pt CNC/CB (**d**–**f**); SAED pattern of a single Pt CNC in Pt CNC/CB projected from the [100] direction (**g**); HRTEM images of Pt CNC/CB (**h**); magnified HRTEM image of the highlighted area in Figure 2h (**i**); atomic model of (510) planes (**j**).

Information related to surface morphology can be provided by TEM measurement. As shown in the low-resolution TEM images for the Pt CNC and Pt CNC/CB samples (Figure 2a,d), uniform nanocubes were successfully synthesized at high yield. It was clearly observed that Pt nanocubes were uniformly dispersed on the CB support, in comparison with the pure Pt CNC sample without catalyst support. The addition of catalyst support can greatly improve the dispersibility of catalysts such that the loss of catalytic activity caused by the aggregation of active sites can be largely avoided [30]. Sufficient exposure of the active surface is beneficial to enhance the catalytic efficiency and improve the catalytic activity of the catalyst. The average sizes of the nanocubes in Pt CNC and Pt CNC/CB were 31.06 ± 0.6 nm and 28.92 ± 0.5 nm, respectively, determined by the edge length (Figure 2c,f).

Figure 2b,e displays the representative HRTEM images of the Pt CNC and Pt CNC/CB samples, from which the concave structures with clear boundaries at the Pt CNC interfaces can be observed. It is well acknowledged that the angles between the facets of the projected concave nanocube and the {100} facets of an ideal cube can be used to deduce the index. Pt showed fcc crystallinity in a monometallic cluster. The selected area electron diffraction (SAED) pattern illustrated that the prepared Pt CNC had high crystallinity along the [100] zone axis (Figure 2g). The surface Miller index was measured on an individual Pt CNC projected along the [100] direction. The angle between the facets of the projected Pt CNC and the {100} facets of an ideal cube was 11°. The theoretical value of the angle between high-index planes with Miller indices (510) and the {100} planes was reported to be 11.3° [15,32]. From the comparison between the measured angles and the ideal values, it is suggested that the as-synthesized Pt CNCs are mainly enclosed by high-index (510) facets. It was reported that the high-index (510) facets can be indicated as [5(100) × (110)] [33]. As shown in Figure 2h,i, the atomic steps on the edge of Pt CNC can be seen clearly, which is consistent with the atomic arrangement of the (510) facets. Figure 2j is an atomic model of the (510) planes in the Pt CNC/CB catalyst, which was established to illustrate the arrangement of atomic steps directly. Such HIFs possess a high density of step atoms, which generally constitute the active sites of electrocatalysts [18].

**Figure 3:**
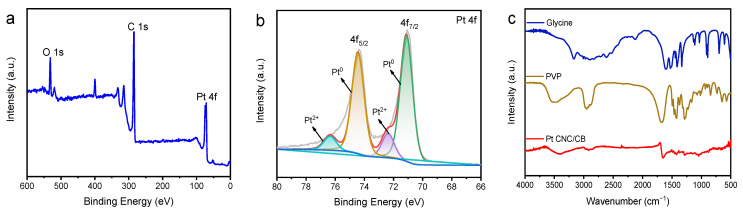
XPS survey spectrum of Pt CNC/CB (**a**); high-resolution XPS spectrum of Pt 4f (**b**); FT-IR spectrum of Pt CNC/CB, PVP, and glycine (**c**).

XPS analysis of Pt CNC/CB was carried out to estimate the chemical state of elements. The binding energy of the C 1s (284.6 eV) was used as the standard for calibration. As shown in Figure 3a, the full survey spectrum showed major peaks for Pt 4f, C 1s, and O 1s at 71.0, 284.6, and 531.4 eV, respectively. The O element mainly originated from the air. The chemical nature of Pt 4f was further analyzed using the deconvoluted XPS spectra as shown in Figure 3b. The Pt 4f peak could be resolved into two pairs of peaks, identified as Pt 4f_7/2_ and Pt 4f_5/2_. The peaks located at around 71.11 and 74.44 eV represent metallic Pt. The peaks at around 72.37 and 76.35 eV can be assigned to the Pt (II) in PtO or Pt(OH)_2_ species [34,35]. The FT-IR spectra of Pt CNC/CB, PVP, and glycine are shown in Figure 3c. The strong characteristic peaks in PVP and glycine could not be observed in Pt CNC/CB, which demonstrates that the redundant PVP and glycine were removed completely after the successful synthesis of the Pt CNC/CB catalyst.

### 3.2. Electrochemical Analysis

**Figure 4:**
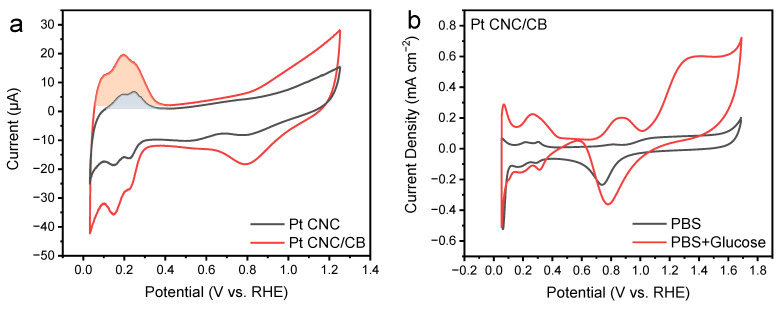
CV curves of Pt CNC/CB and Pt CNC in a 0.5 M H_2_SO_4_ solution (**a**); CV curves of Pt CNC/CB in 0.1 M PBS (pH 7.4) solution with and without 50 mM glucose (**b**).

The ECSA of catalyst is associated with the charges in both the adsorption and desorption processes of hydrogen [36]. The ECSA value is evaluated by integrating the peak area of the CV scanning, performed in a nitrogen-saturated 0.5 M H_2_SO_4_ solution with a scan rate of 50 mV s^−1^. The charge was normalized using a surface area-specific charge of an ideal one-electron transfer, which is 210 μC cm^−2^ [37,38]. As shown in Figure 4a, the peak area of Pt CNC/CB was larger than that of the Pt CNC catalyst. The calculated ECSA value of the Pt CNC/CB catalyst was 2.80 m^2^ g^−1^, which was approximately two times higher than that of Pt CNC (1.56 m^2^ g^−1^). The higher specific ECSA of Pt CNC/CB implies a greater dispersion of Pt CNCs, which is beneficial to the exposure of the HIFs of Pt CNCs. The HIFs have a high density of low-coordinated atoms, which can serve as highly active sites to break C–H bonds in glucose oxidation reactions.

Figure 4b shows the catalytic performance of the Pt CNC/CB anode in the glucose oxidation reaction. The CV test was evaluated in 0.1 M PBS (pH 7.4) with 50 mM glucose electrolyte. A CV test in 0.1 M PBS (pH 7.4) solution without the addition of glucose was also conducted for comparison. As shown in Figure 4b, obvious peaks could be observed from the CV curve conducted in the 0.1 M PBS (pH 7.4) with 50 mM glucose electrolyte, which demonstrates the oxidation reaction of glucose. During the oxidation process, the glucose is first electrooxidized to gluconolactone and then hydrolyzed to gluconic acid [39,40]. The electrocatalytic oxidation of glucose can be characterized by the peaks at three potential ranges. The oxidation peak in the hydrogen region (0.15–0.35 V) demonstrates the adsorption of glucose molecules on the surface of the Pt CNC/CB anode. The bond breaking between the hemiacetal carbon and the attached hydrogen atom is considered as the rate-limiting step in the glucose electrooxidation reaction [9,41,42]. In this step, both the glucose molecule and the hydrogen atom are adsorbed on the electrode surface through the Pt CNC/CB catalyst (Equations (1)–(3)).

(1) $$\mathrm{Pt}+{\;C}_{6}H_{12}O_{6}\;\to \mathrm{Pt}{\left(C_{6}H_{11}O_{6}\right)}_{\mathrm{ads}}+H$$

(2) Pt + H → Pt−H

(3) $$\mathrm{Pt}-H\;\to \;\mathrm{Pt}\;+{\;H}^{+}+{\;e}^{-}$$

In the double-layer region (0.4–1.0 V), as the applied potential increases, abundant OH_ads_ species are formed through the fast decomposition of water. According to the ‘Incipient Hydrous Oxide Adatom Mediator’ model (IHOAM) proposed by Burke, the incipient hydrous oxide layer of OH_ads_ is generated through a premonolayer oxidation step on the surface of active metals, which can mediate the oxidation of glucose [43,44]. The active OH_ads_ layer is more likely to generate on the active sites with low lattice coordination values and disconnected areas such as the edges. Thus, the concave structure in the Pt CNC/CB catalyst is considered to be a favorable structure that can serve as an active site for the electrocatalytic oxidation of glucose (Equations (4)–(6)).

(4) $$H_{2}O\;\to {\mathrm{OH}}_{\mathrm{ads}}+{\;H}^{+}+{\;e}^{-}$$

(5) $$C_{6}H_{11}O_{6}+{\left(\mathrm{OH}\right)}_{\mathrm{ads}}\to C_{6}H_{10}O_{6}+H_{2}O$$

(6) $$C_{6}H_{10}O_{6}+H_{2}O\;\to {\;C}_{6}H_{12}O_{7}$$

A peak located at the potential over 1.1 V was observed, which is regarded as the oxygen region. In this region, the active OH_ads_ species on the surface of Pt CNC/CB are stripped and replaced by the O_ads_ species, which exhibit less activity than OH_ads_. The O_ads_ adsorbed on the surface of the Pt CNC/CB catalyst facilitate the formation of the PtO layer, which can oxidize the glucose molecule directly (Equations (7) and (8)) [40]. The peak current densities measured on the Pt CNC/CB anode were 0.22, 0.20, and 0.60 mA cm^−2^. It exhibited higher catalytic activity than Pt CNC and commercial Pt/C catalysts compared with our previous study [29]. The improved catalytic activity can be attributed to the addition of CB support, which largely improved the dispersity of the Pt CNCs and allowed adequate exposure of the active HIFs of Pt CNCs.

(7) $$C_{6}H_{12}O_{6}+\;\mathrm{PtO}\;\to {\;C}_{6}H_{10}O_{6}+\;\mathrm{Pt}\;+{\;H}_{2}O$$

(8) $$C_{6}H_{10}O_{6}+{\;H}_{2}O\;\to C_{6}H_{12}O_{7}$$

**Figure 5:**
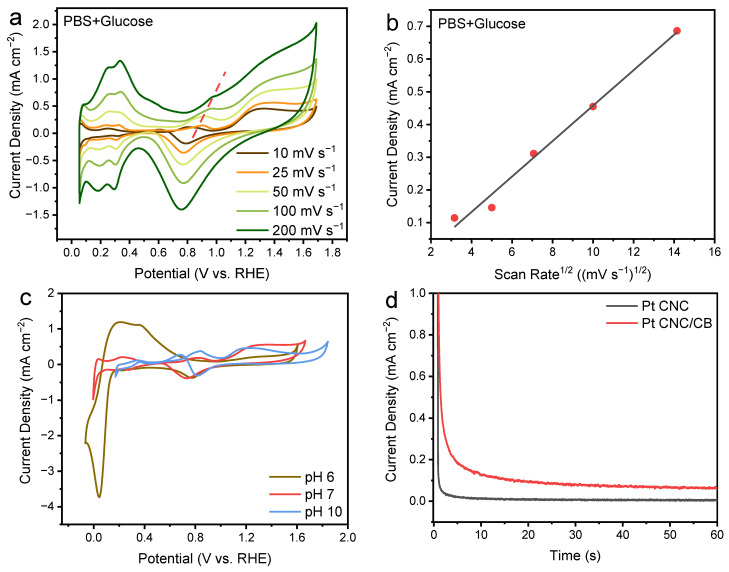
CV curves of Pt CNC/CB in the 0.1 M PBS (pH 7.4) and 50 mM glucose electrolyte at different scan rates (v = 10, 25, 50, 100, and 200 mV s^−1^) (**a**); scan rate dependence of the current density for Pt CNC/CB in the 0.1 M PBS (pH 7.4) solution with 50 mM glucose (**b**); CV curves of Pt CNC/CB in the 0.1 M PBS solution with 50 mM glucose at different pH values (pH = 6, 7, and 10) (**c**); CA curves of the Pt CNC/CB electrode at 1.279 V in the 0.1 M PBS (pH 7.4) and 50 mM glucose electrolyte (**d**).

As shown in Figure 5a, a series of CV curves of the fabricated Pt CNC/CB anode were measured by varying the scan rate from 10 mV s^−1^ to 200 mV s^−1^ in a mixing solution of 0.1 M PBS (pH 7.4) and 50 mM glucose. Based on the CV curves, as the scan rate increased, the peak current densities presented an upward trend. Figure 5b shows the linear relationship between the current density of the oxidation peak and the square root of the scan rate, which reveals the typical diffusion-controlled electrochemical behavior on the Pt CNC/CB anode [11].

The catalytic performance of the Pt CNC/CB electrode toward glucose oxidation in the mixing solution of 0.1 M PBS and glucose was detected at different pH values. The pH values 6, 7, and 10 were selected on behalf of the acidic, neutral, and basic conditions. From Figure 5c, the CV curve detected in the solution of pH 6 exhibited the highest peak current density among all pH values in the hydrogen region, which can be associated with adequate hydrogen ions in the acidic electrolyte. However, in the double-layer region, the peak current density under the basic conditions was much more obvious than that in the acidic and neutral solutions. In the basic environment, abundant OH^−^ are likely to be adsorbed on the surface of the Pt CNC/CB electrode, which can serve as active sites for the glucose oxidation reaction. With the increase in the applied potential, the active OH_ads_ are replaced by O_ads_ in the oxygen region. The peaks observed in the basic and neutral electrolyte in the oxygen region were higher than those under the acidic conditions. Therefore, the electrocatalytic process of the Pt CNC/CB electrode varies with different pH values of the electrolyte [44].

The stability of the Pt CNC/CB and Pt CNC electrodes in the glucose oxidation reaction were further evaluated by CA tests at 1.279 V for 60 s. As shown in Figure 5d, the CA curves demonstrated a fast decrease initially and then became stable. After 60 s, the steady current densities of glucose oxidation on the Pt CNC/CB and Pt CNC electrodes were 0.061 mA cm^−2^ and 0.0062 mA cm^−2^, respectively. This reveals that the Pt CNC/CB electrode exhibited higher activity and stability compared to the Pt CNC electrode. The loading of Pt CNCs on the CB catalyst support may be beneficial, effectively facilitating the exposure of the active HIFs of Pt CNCs.

Compared with the conventional Pt nanoparticle catalysts, the catalytic activity of Pt CNC was greatly improved by a high density of atomic steps provided by the HIFs on the surface of Pt CNCs, which served as active sites in the glucose oxidation reaction. During the catalytic process, the discontinuous structures with low coordination numbers are more likely to adsorb or bond with the glucose, OH_ads_, O_ads_, and the intermediates. The addition of the CB catalyst support effectively increased the dispersion of Pt CNCs, which facilitated the full exposure of the active structures. The sufficient exposure of active sites improved the catalytic efficiency and thus enhanced the catalytic activity of Pt CNC/CB. Moreover, the stability of Pt CNC/CB was strengthened by coating the Pt CNCs on the CB catalyst support with a large surface area, which can effectively avoid the agglomeration of the Pt CNCs.

## 4. Conclusions

In summary, a Pt CNC/CB catalyst was synthesized using a facile hydrothermal method. The prepared catalyst presented high catalytic activity and good stability for the electrocatalytic oxidation of glucose under neutral conditions. Reasons for the enhanced catalytic properties can be expressed as follows. First, the CB catalyst support provided a large surface area for the sufficient dispersion of Pt CNCs, which facilitated the full exposure of the active HIFs. Second, the good chemical stability and remarkable mechanical properties of the carbon material helped to improve the stability of the Pt CNC/CB catalyst. Third, the high densities of atomic steps, edges, and kinks derived from the HIFs of Pt CNC were favorable for boosting the electrocatalytic activity of the Pt CNC/CB catalyst. This work provides an alternative catalyst for further application in implantable glucose fuel cells and electrochemical biosensors, which are capable of power supply and glucose detection in the human body.

## Data Availability

The data presented in this study are available on request from the corresponding author.
